# Community caregivers’ perceptions: Family-centred postnatal care in KwaZulu-Natal, South Africa

**DOI:** 10.4102/phcfm.v17i1.4817

**Published:** 2025-04-29

**Authors:** Nontuthuzelo D. Nyasulu, Myra Taylor, Nelisiwe Khuzwayo

**Affiliations:** 1 Discipline of Public Health Medicine, School of Nursing and Public Health Medicine, College of Health Science, University of KwaZulu-Natal, Durban, South Africa

**Keywords:** family centred care, family centred postnatal care, postnatal care, community care giver, saving mothers, saving babies

## Abstract

**Background:**

The family-centred postnatal care (FCPC) approach highlights the active participation of family members in supporting the care of the mother and newborn. It acknowledges the vital role of social support, practical assistance and emotional encouragement from family members.

**Aim:**

The study aimed to explore community caregivers’ (CCGs) experiences and perceptions of the FCPC approach.

**Setting:**

The study took place in uMgungundlovu municipality, KwaZulu-Natal Province.

**Methods:**

A qualitative study design using a grounded theory approach was used. Participants were selected using purposive sampling. Four focus group discussions were held with 23 CCGs from the seven sub-districts. Data were analysed manually following the grounded theory steps.

**Results:**

Five themes emerged from the data analysis. These include perceptions of CCGs about the strength of antenatal care in enhancing postnatal care (PNC), experiences of CCGs in FCPC, non-attendance of postnatal visits by mothers and newborns, attitudes of CCGs towards healthcare professionals, and recommended activities for the CCGs in FCPC.

**Conclusion:**

The data analysis highlights the vital contributions of CCGs in improving maternal health and neonatal outcomes. These findings underscore the critical areas for improving support and outcomes for mothers and newborns.

**Contribution:**

The experiences and perceptions of CCGs on FCPC provide valuable insights into the importance of involving family members in supporting mothers and newborns. Their perspectives highlight practical challenges, opportunities for improved care, and family engagement’s critical role in enhancing postnatal outcomes.

## Introduction

Postnatal care (PNC) is recognised as a global critical component of maternal and child health (MCH) services.^1^ The postnatal period, which encompasses the first 6 weeks after childbirth, is a vulnerable time for both the mother and the newborn, requiring careful attention to prevent complications such as maternal infections, postpartum haemorrhage, neonatal sepsis and other life-threatening conditions.^2^ The World Health Organization (WHO) highlights the significance of PNC in lowering maternal and newborn mortality rates, especially in low- and middle-income countries (LMICs), where access to high-quality healthcare services may be restricted.^3^ In South Africa, maternal and neonatal mortality rates have declined recently; for instance, maternal mortality in facility ratio (MMFR) decreased from 105.9 deaths per 100 000 live births in 2019 to 88.0 in 2020.^4^ Conversely, neonatal mortality rates have also improved, though specific recent figures are limited. Maternal health indicators in KwaZulu-Natal have exhibited notable trends in recent years. The rate of first antenatal visits before 20 weeks slightly increased from 74.4% in 2019 to 74.6% in 2020. The Caesarean section delivery rate rose from 32.0% in 2018 to 35.0% in 2020. Encouragingly, the maternal mortality ratio in health facilities declined from 101.9 deaths per 100 000 live births in 2018 to 76.9 in 2020. The stillbirth rate in facilities remained stable at 22.0 per 1000 births between 2018 and 2020. In 2020, neonatal mortality in facilities was recorded at 11.5 deaths per 1000 live births, with early neonatal mortality at 9.0 per 1000 live births.^5,6^

Family-centred postnatal care (FCPC) is one strategy that has gained increasing attention in improving MCH outcomes.^7,8,9^ This approach emphasises the active involvement of family members in the care of the mother and newborn, recognising that social support, practical assistance and emotional encouragement from family members play a critical role in the recovery and health of postpartum women and their infants. Family-centred care (FCC) is based on the understanding that families are the constant in a person’s life, while healthcare professionals and systems can change over time.^10,11^ Healthcare systems should improve communication, enhance maternal and newborn well-being and foster stronger family bonds by incorporating family members into PNC.

In the context of South Africa, where socio-economic disparities, cultural diversity and varying access to healthcare services exist, the role of FCPC becomes even more critical. Families postnatally serve as primary caregivers, especially in rural areas with scarce healthcare resources.^8,9,12,13^ However, the extent to which FCC is effectively integrated into postnatal services remains unclear. Various factors, including cultural beliefs, healthcare system limitations and the capacity of healthcare providers, may influence the success of family-centred approaches in South Africa.^14,15^ Moreover, community caregivers (CCGs), often tasked with providing postnatal support at the grassroots level, play a crucial role in ensuring that FCPC is implemented effectively.^15,16^ Therefore, their perceptions, experiences and insights are invaluable in understanding the practical challenges and opportunities in delivering FCPC.

Community caregivers in South Africa are an essential link between the formal healthcare system and the community, particularly in rural and underserved areas.^16,17^ These caregivers, often women from the local communities, provide various services, including health education, essential medical assistance and psychosocial support. In the postnatal context, CCGs are often the primary source of support for new mothers, particularly those who may not have easy access to healthcare facilities.^16,18^ They guide breastfeeding, hygiene, immunisations and newborn care while offering emotional support to mothers adjusting to their new roles.^19,20,21^ Given their close interactions with families, CCGs are well-positioned to offer valuable insights into the functioning of FCPC in diverse settings.

Despite CCGs’ critical role in PNC, limited research explores their perceptions of family-centred approaches. Community caregivers’ perspectives on family involvement, their challenges and the factors that facilitate or hinder FCC are essential for informing policy and programmatic decisions. In addition, the insights on CCGs can contribute to developing tailored interventions that enhance PNC delivery in South Africa. The existing literature on FCC in South Africa tends to focus on hospital-based interventions, leaving a gap in understanding the role of community-based care.^22,23^ In South Africa, ongoing efforts have been to address MCH challenges, especially in rural areas; exploring CCGs’ perspectives on FCPC is timely and necessary. This study seeks to fill this gap by examining the experiences and perceptions of CCGs in South Africa, thereby providing a deeper understanding of how FCC is delivered at the community level and identifying areas for improvement in MCH services.

## Research methods and design

### Study design

A qualitative interpretive approach, employing a grounded theory design, was used to explore CCGs’ experiences and perceptions of FCPC.^23^

### Setting

The study was conducted in South Africa, KwaZulu-Natal province, in uMgungundlovu District, a largely rural district. This district has a population of 1 052 730, of whom the majority (79%) are isiZulu speaking, and 52% are women. The selection of this district was also influenced by the age of young mothers who give birth in the facilities. Women between 10 and 24 years constitute about 30.0% of the population, while 26.1% of women between 10 and 19 years have given birth in one of the local hospitals. Given the young age of women giving birth in this district, the authors consider that young mothers will benefit from FCPC as they need continued PNC, including breastfeeding and reproductive health care support.

### Study population and sampling strategy

The study population consisted of CCGs residing in a district municipality, specifically those affiliated with local primary health care (PHC) clinics providing PNC. Non-probability purposive sampling was used to select 23 CCGs employed by the Department of Health and conducted household visits in their communities. The saturation principle was applied to determine the study’s sample size. Recruitment focussed on clinics with attached CCGs, targeting information-rich participants with direct experience with FCPC. The sample represented CCGs from seven local subdistrict municipalities within the district. Participants were included only if they were attached to a local clinic; mothers were included if they had newborns, and any family members who cared for a mother and the baby could not participate in the study.

### Data collection

Data were collected through four focus group discussions (FGDs) held from 22 May to 06 June 2023. An interview guide was written in English, translated into isiZulu, and back-translated into English. Before data collection, the interview guide was piloted to ensure that the questions answered the study’s research question. During data collection, the researcher explained the study’s purpose, and participants signed consent forms, with anonymity ensured by assigning codes. Participation was voluntary, and permission was obtained to use an audio recorder. The discussions, led by the principal author and a second author who observed non-verbal cues, lasted 45 min to an hour, and the FGDs were conducted in isiZulu. The FGDs were conducted in private rooms within the local clinics. These FGDs were conducted in local clinics because participants felt clinics were neutral venues accommodating their travelling distances.

### Data analysis

Data collection and analysis occurred concurrently, allowing the researchers to adjust the questioning and further probe into emerging concepts as themes developed.^24^ After a FGD, the data were transcribed verbatim and translated from isiZulu to English before being analysed manually.

The authors constantly compared similarities, differences and patterns within the data.^25^ Coding was conducted in phases, starting with open coding, followed by axial and selective coding to categorise data into key themes representing caregivers’ FCPC experiences.^26^ This iterative process ensured that the theory emerged directly from the caregivers’ insights, leading to a grounded theoretical model that explains how CCGs understand and navigate FCPC in the South African context.

### Trustworthiness

This qualitative study utilised FGDs to gather in-depth insights. Credibility was established through the systematic use of original data, with verbatim quotes in the results section to represent participants’ perspectives accurately.^27^ Peer debriefing sessions were held after each discussion by the authors and data collector to enhance credibility further. Confirmability was ensured by assessing how well the findings aligned with the discussions and that the analysis was firmly grounded in the data.^27^ The research questions guided the coding process. All records were securely stored in a locked cupboard, accessible only to the researcher. To validate the findings, the researcher engaged participants by providing feedback on emerging data and interpretations, ensuring that the final analysis reflected their views accurately.^28^ An inductive approach allowed the data to shape the themes. Transferability was supported through thick descriptions, enabling readers to determine if the findings could apply to other contexts.^28^ Dependability was maintained by conducting an audit trail with the authors, ensuring the analysis was consistent, grounded in the data and stable over time.^28^

### Ethical considerations

Ethical clearance to conduct this study was obtained from the University of KwaZulu-Natal (UKZN) Biomedical Research Ethics Committee (No. BREC /00004882/2022). Permission was received from the Health Research Committee of KwaZulu-Natal Provincial Department of Health. No monetary reimbursement was given to the participants; however, each group was offered a light lunch during each FGD. Before the discussions, the researcher emphasised the importance of confidentiality of the information shared within the group. It was also explained that the transcripts would be anonymised and that records would be kept strictly confidential.

### Reflexivity

Throughout this study, the first author navigated between the roles of an indigenous insider and an indigenous outsider.^29^ As an indigenous insider, her background as a midwife, national maternal health trainer and master’s degree holder provided valuable insight into the internal workings of CCGs, enabling her to facilitate discussions about caregiving experiences with participants. However, because she was not a CCG, she could not fully grasp the personal and subjective aspects of caregiving, positioning her as an indigenous outsider.^29^

Foregrounding the inherent tensions between these researcher subject positions allowed me to engage with the boundaries of the phenomenon under study, which is my understanding of caregiving during postnatal care. More specifically, my identity as an indigenous insider and indigenous outsider influenced how I related to the scientific aspects of the research study, such as the phrasing of the study aim and research question, the study approach and how the data were analysed and produced. My acknowledgement of the ‘self as the instrument’, which is central to qualitative research, meant that the voice accorded to ‘the other’ (participants) can only be described as partial in my presentation and discussion of the interview transcripts in this paper. Notwithstanding the above-stated comments, during this research, I always attempted to centralise the lives of my participants. I was always aware that I may have inadvertently traumatised them through their participation in this study. In this regard, CCGs who participated in this study did so voluntarily; I also made myself available after the completion of this study to provide feedback regarding the study results.^29^

## Results

### Socio-demographic characteristics of the participants

Table 1 highlights the socio-demographic characteristics of the 23 CCGs participating in the FGDs. Each FGD consisted of five to six participants, ages 23–60 years. There were 22 (96.6%) females, 1 (4.3%) male participant, and 73.9% had completed school in Grade 12.

**TABLE 1:** Socio-demographic characteristics of the participants.

Participants	Number of participants	Age range of participants (years)
FGD 1	06	23–46
FGD 2	06	23–38
FGD 3	05	26–51
FGD 4	06	33–60

**Total**	**23**	**23–60**

FGD, Focus Group Discussion.

Five key themes emerged from the data analysis. The themes include: (1) Perceptions of CCGs on the strength of antenatal care (ANC) in enhancing PNC, (2) Experiences of CCGs in FCPC, (3) Non-attendance of postnatal visits by mothers and newborns, (4) Attitudes of CCGs towards healthcare professionals, and (5) Recommended activities for the CCGs on family-centred approach in PNC.

### Perceptions of community caregivers on the strength of antenatal care in enhancing postnatal care

Participants highlighted the intrinsic connection between both ANC and PNC in improving maternal and neonatal health outcomes, emphasising that this linkage must be prioritised to effectively implement a family-centred approach in PNC. They noted that preparing mothers during pregnancy is essential for fostering positive health outcomes for both mothers and their babies, and emphasised that CCGs play a pivotal role in ensuring continuity of care. Participants stressed that as CCGs, they encourage pregnant women to attend all recommended ANC visits, which allow for early identification and management of potential complications. Their home visits also provide opportunities for CCGs to educate mothers about PNC, emphasising the role of family support in maintaining consistent health practices and addressing postpartum challenges. Integrating families in PNC strengthens this continuum by fostering a supportive maternal and neonatal recovery environment. Postnatal care must follow the foundation established by the timeous initiation of prenatal care. Antenatal care is essential to identify potential problems and to monitor the health of both mother and child throughout the pregnancy to provide the information needed to prepare for delivery and to reduce potential risks. Postnatal care is closely linked to prenatal care, as it is affected by any complications that may be identified during antenatal visits. Problems such as diabetes, hypertension or preterm labour need to be identified early and monitored to reduce complications during delivery or thereafter:

‘It is not easy to talk about postnatal care without talking about prenatal care because prenatal care establishes a foundation for monitoring the mother’s and baby’s health, addressing risks, and preparing for delivery, which directly influences postnatal outcomes. Effective prenatal care reduces complications that may arise during or after childbirth.’ (Participant 1, 26 year old female, FGD 2)

### Experiences of community caregivers in family-centred postnatal care

Participants mentioned that CCGs already work closely with families in various health services, playing an essential role in promoting maternal and newborn health. They described how CCGs assist families and mothers of newborns by monitoring the mother’s physical recovery, such as healing after delivery and addressing emotional well-being, including signs of postpartum depression or anxiety. Community caregivers also focus on ensuring the newborn’s health through regular check-ups, identifying potential issues such as low birth weight or infections, and educating parents about immunisation schedules:

‘As CCGs, we support families and mothers of newborns by overseeing the mother’s physical recovery, including post-delivery healing, and addressing emotional health by identifying signs of postpartum depression or anxiety. We also prioritise the newborn’s well-being through routine check-ups, early detection of concerns like low birth weight or infections, and educating parents on immunisation schedules.’ (Participant 3, 50 year old female, FGD 1)

In addition, participants noted the critical role of CCGs in guiding breastfeeding and nutrition, emphasising the importance of exclusive breastfeeding during the first 6 months and introducing appropriate complementary foods. Community caregivers were seen as instrumental in this phase through home visits and regular follow-ups, which help address postpartum challenges such as fatigue, breastfeeding difficulties or the lack of support from family members:

‘CCGs can observe and assess the mothers’ and babies’ condition to ensure they are well cared for and not given solid food before they complete six months. If the family is also taught, they can assist her (the mother), help her with things like baby bath and preparing meals for her, to allow the mother to rest.’ (Participant 1, 29 year old female, FGD 4)

Participants also highlighted how CCGs facilitate open communication within families, encouraging shared responsibilities in caregiving. By involving family members, CCGs promote a supportive environment that eases the mother’s transition into postpartum care and ensures a stable, nurturing setting for the newborn’s development. Through these efforts, CCGs bridge the gap between healthcare facilities and the community, fostering holistic care for mothers and babies:

‘We check and teach the mothers and family about the care of the woman’s stitches if she had caesarean and episiotomy.’ (Participant 2, 25 year old female, FDG2)

‘We teach the family and the mother how to recognise signs of abnormal vaginal bleeding and be aware of soaked sanitary pads and heavy big clots. We work with health professionals when necessary; we refer those mothers to the clinic for further postpartum care management.’ (Participant 3, 47 year old female, FGD 4)

### Non-attendance of postnatal visits by mothers and newborns

Participants indicated that some mothers, particularly teenagers, tend to avoid seeking postnatal visits to the clinic for care, a behaviour that the CCGs believe significantly contributes to adverse postnatal outcomes. This reluctance to attend postnatal clinics may be attributed to various factors. Fear of being judged by healthcare professionals, family members or the broader community often discourages young mothers, especially those facing stigma because of early or unplanned pregnancies:

‘The reluctance of pregnant mothers to attend postnatal clinics can stem from multiple factors. Young mothers, particularly those dealing with stigma from early or unplanned pregnancies, often feel discouraged by fear of judgment from healthcare professionals, family members, or the wider community.’ (Participant 4, 50 year old female FGD 2)

In addition, a lack of knowledge about the importance of early medical intervention in addressing potential health complications for both the mother and the newborn further contributes to their avoidance. Some mothers may not fully understand the risks of neglecting PNC, such as untreated postpartum infections, breastfeeding challenges or undiagnosed neonatal health issues:

‘Some mothers lack knowledge of the consequences of the complications of pregnancy and post-delivery care, and others who are not pregnant for the first time are ignorant.’ (Participant 5, 30 year old female, FGD1)

Moreover, participants noted that social pressures and cultural factors could affect this behaviour. For instance, some mothers, particularly teenagers, might wish to avoid revealing their baby’s existence to friends or community members because of fear of gossip, criticism or shame. This desire for privacy and limited access to transportation or childcare for their older children can create significant barriers to attending postnatal visits. Community caregivers emphasised the urgent need to address these challenges by fostering a non-judgemental and supportive environment at clinics, increasing community awareness about the value of PNC and ensuring that healthcare services are accessible and accommodating to the unique needs of mothers:

‘These young school-going girls hide the pregnancy because of fear of being judged by their families … Community members’ CCGs are in a position to identify pregnancy since we are based [*in*] the communities.’ (Participant 1, 57 year old female, FGD 1)

‘The older women also give us problems because they have given birth before they think that they do not need ANC, and as a result, they tend to come to the clinic just once to book the bed for delivery.’ (Participant 5, 35 year old male, FGD 2)

### Recommended activities for the community caregivers on family-centred approach in postnatal care

Almost all the participants highlighted that CCGs play a crucial role in the post-delivery care period, particularly at the household level. They emphasised that CCGs are well-positioned to monitor the health and recovery of the mother and baby during this critical time. One key responsibility identified by the participants is the CCGs’ ability to observe and assess the mother’s condition, particularly postpartum bleeding, ensuring that any signs of complications are promptly addressed:

‘We are in an excellent position to closely monitor the health and recovery of both the mother and baby during the crucial period after birth. By the nature of our operation, our role includes observing and evaluating the mother’s condition, particularly regarding postpartum bleeding, to ensure any signs of complications are identified and addressed without delay.’ (Participant 4, 46 year old female, FGD 4)

In addition, CCGs must be responsible for monitoring the baby’s health, with particular attention to the umbilical care, as proper care of the umbilical cord is essential for infection prevention and healthy healing. They should also guide mothers and family members on dressing the baby to promote the healing of the umbilical navel:

‘As CCGs, we also examine how the baby’s navel heals and tell her (the baby’s mother) how to dress the baby if the navel has not yet healed. We advise her to use the clothes that will give fresh air for the baby’s navel to heal, not the clothing that will cover the navel.’ (Participant 2, 42 year old female, FGD 3)

Furthermore, CCGs must play an important educational role by advising mothers on best breastfeeding practices and ensuring that babies receive proper nutrition. They also guide mothers on when to introduce solid foods, ensuring the baby’s smooth and healthy transition:

‘We make sure that mothers know every thing about the newborn, for instance how to breastfeed, when to introduce solid food and the type of food that is suitable for the baby so that the baby can be healthy.’ (Participant 1, 29 year old female, FGD 4)

This comprehensive support provided by CCGs during the postnatal period contributes significantly to the well-being of both mother and child, as highlighted by the participants.

### Attitudes of community caregivers towards healthcare professionals

Some participants highlighted the behaviour of mothers during pre- and post-delivery, which often creates tensions between the CCGs and healthcare professionals. They explained that this tension arises because some mothers fail to adhere to ANC and PNC recommendations, which health professionals interpret as a failure by the CCGs to fulfil their responsibilities effectively. Nurses and healthcare leadership may mistakenly perceive CCGs as neglecting their duties to identify, support, and refer mothers and their babies to healthcare facilities for essential care:

‘The nurses and the leadership are also not impressed with CCGs regarding poor ANC visits, especially among adolescents. They feel we fail to carry out our duties.’ (Participant 5, 44 year old female, FGD 4)

Another participant said:

‘When mothers do not honour post-delivery visits or arrive with the sick neonate, some CCGs are blamed by the health professionals.’ (Participant 5, 33 year old female, FGD 1)

Participants pointed out that the misconceptions often fail to account for the significant challenges CCGs encounter when trying to engage mothers who may resist seeking care because of stigma, cultural norms or socio-economic difficulties. For example, some mothers may avoid clinics to keep their pregnancies or births hidden. In contrast, others face obstacles such as limited transportation or the lack of family support, making it challenging for CCGs to maintain consistent follow-ups:

‘… CCGs face challenges when conducting home visits; there are things beyond their control, for example, some do not have transport fees, and others do not have family support.’ (Participant 5, 38 year old female, FGD 3)

Participants stressed the need for better alignment and mutual understanding between CCGs and healthcare professionals. They suggested regular meetings, joint training sessions and transparent communication as critical steps to foster collaboration, clarify roles and create a unified approach to supporting mothers and their babies. This would ease tensions and enhance the effectiveness of maternal and PNC services in the community:

‘… for successful implementation of the Family postnatal care approach, the leadership must make investments to promote collaboration between health professionals and CCGs.’ (Participant 5, 38 year old female, FGD 3)

## Discussion

The study aimed to explore the experiences and perceptions of CCGs on the FCPC approach in uMgungundlovu District Municipality, KwaZulu-Natal province. The study’s findings confirmed the involvement of CCGs in the FCPC. Drawing on their experiences, CCGs often emphasise personalised support tailored to the specific needs of mothers and newborns. They address non-attendance at postnatal visits by proactively engaging families, educating them on the importance of these check-ups and facilitating access to healthcare services. These results are similar to studies on the roles of CCGs conducted in South Africa.^2,9,30^

This study’s findings reaffirm that PNC is intrinsically linked to ANC, as the mother’s and child’s health outcomes largely depend on consistent attendance at all recommended healthcare visits during and after pregnancy.^31,32^ This highlights the continuity of care required for optimal maternal and newborn health outcomes. The research underscores the pivotal role of CCGs in enhancing the implementation of the FCPC. Through their collaboration and partnerships with healthcare providers, CCGs provide essential support at the household level, particularly in developing strategies to care for newborns.^18^

Positioned within their communities,^33^ CCGs have a unique ability to identify and address various health-related issues.^31^ In this study, participants confirmed that the CCGs deliver PNC services at the household level. They described their roles as providing education and awareness about maternal care and PNC, identifying early pregnancies, and promoting and supporting the mother and baby in maternal care and PNC. The audits conducted by the ‘South African Every Death Counts Writing Group’ highlight preventable factors associated with postnatal maternal and newborn deaths, underscoring the critical need for addressing gaps in care.^2,34^ These audits emphasise empowering families to demand quality healthcare services actively. This has significant implications for the role of CCGs and highlights the relevance and importance of their interactions with families and communities.

One of the key responsibilities of CCGs is to ensure that families are equipped to advocate for and demand quality care.^8,9,35^ However, families may struggle to know what to ask for if they are not adequately informed. To address this gap, CCGs confirmed in this study that they provide mothers and their families with relevant, accessible healthcare information, particularly about danger signs during the postnatal period and the importance of timely care-seeking behaviour.^36^ By delivering these key health messages, CCGs can empower families to recognise when something is wrong and seek help promptly, thereby demanding and improving the quality of their proper care.

At the household level, improving the mother and child’s health outcomes depends on collaboration and cooperation between pregnant women, mothers of newborns and their family members.^8,12^ The study results show that although CCGs play a role in educating communities on maternal and postnatal services, participants reported challenges that continue to exist, such as poor attendance at ANC visits.^37^ Young mothers miss out on critical health assessments and interventions that are essential for ensuring both their well-being and that of their unborn child.^37^ Community caregivers expressed concern that this avoidance behaviour can lead to severe complications, such as stillbirths or maternal bleeding during delivery, which could have been prevented with proper medical care.

By not attending antenatal visits, teenagers and older pregnant women increase their risk of experiencing poor pregnancy outcomes, placing both their lives and the lives of their babies in jeopardy.^30^ Without timely referrals to antenatal clinics, the risks of complications during pregnancy and childbirth, such as stillbirths or maternal health issues, are significantly heightened.^14,36^ Community caregivers find themselves walking a fine line between honouring the trust of young women and fulfilling their duty to ensure that medical care is provided promptly. This tension complicates their work and has potential long-term consequences for the health outcomes of both the mother and the baby. Community caregivers see their role as vital in addressing this issue by encouraging pregnant women to attend ANC, but they recognise the barriers that complicate these efforts. The other challenges entail difficulty in the identification of pregnancy, in particular for school-going girls. According to the participants, pregnancy is hidden until one is due for delivery, owing to fear of being labelled and discriminated against. However, it is not only among young women, but interestingly, previous studies show that because of social, spiritual and cultural factors, women would only disclose their pregnancy after the first trimester.^37,39,40^

Linking communities with healthcare services is a vital aspect of FCPC, as it fosters a more integrated and accessible health system for families, especially during the critical postnatal period.^41^ Community caregivers are central to bridging this gap, ensuring that families receive timely and appropriate care following childbirth.^42^ Community caregivers are beneficial because they are embedded within the community, know the families and can identify and address health needs early, facilitating direct referrals to healthcare facilities and professionals.^20^ This connection is crucial for ensuring that mothers and newborns receive the follow-up care necessary for their well-being, including monitoring for complications, offering guidance on breastfeeding and supporting family members in caring for the newborn.^8,16,43^ The involvement of CCGs in FCPC strengthens the continuity of care. It promotes a holistic approach to postnatal health, where the family unit is actively engaged in the care process.^8,9^ This close link between community services and PHC facilities helps reduce barriers to care, such as transportation or clinic access issues. It ensures that families, particularly in underserved areas, can access the support they need for a healthier postnatal experience.^34^

Community caregivers in this study elaborated on how the referral process works, noting that CCGs typically write formal referral letters or make direct, face-to-face referrals to healthcare professionals. For instance, when CCGs participate in MCH awareness campaigns, they often encounter clients who require medical attention. In such cases, they refer these clients directly to the professional nurses present at the event. To ensure continuity of care, CCGs also conduct follow-ups with the clinic to learn about their referral’s outcome and confirm that the necessary healthcare services were provided. This hands-on approach demonstrates CCGs’ proactive role in facilitating healthcare access. However, participants believe that enhancing the system to involve families would increase the efficiency and effectiveness of community health management.^44^

Community caregivers can bring valuable experience and knowledge to inform other FCPC team members. During the FGDs, we observed that CCGs actively provided health promotion and health education but less practical skills. This may be a gap in the CCG training modules, which should include practical skills training, thus enabling them to use practical demonstrations to illustrate what they teach. With practical skills, CCGs can demonstrate techniques such as Kangaroo Mother (KMC) to ensure the baby is kept warm, bathing and massaging the abdomen to expel clots after birth. These are essential skills to teach mothers and family members.

An FCPC model is needed to emphasise the collaboration between healthcare providers, CCGs, mothers and family members. For this to be implemented, a policy on FCPC is required in terms of the reorientation of health care providers, including CCGs, and involving communities and families to ensure a clear understanding of FCPC and its implementation.^38^

## Limitations of the study

The study was conducted with a limited number of CCGs within a specific geographical area. As a result, the findings may not represent all CCGs across South Africa or other regions. This limits the transferability of the results to a broader context. Although this was clarified before the data collection, CCGs may have provided socially desirable answers, especially if they perceived that their responses could affect their work evaluations or relationships with healthcare systems.

## Recommendations

Future studies should involve a larger, more diverse sample of CCGs from different provinces and regions to enhance the generalisability of the findings. Comparative studies across different healthcare settings may also help to identify best practices for implementing the FCPC approach. The findings suggest that CCGs would also benefit from additional training on the FCPC approach. Continuous professional development, coupled with peer support groups, could enhance their capacity to deliver high-quality care and address the evolving needs of families during the postnatal period. The success of the FCPC approach requires collaboration between CCGs, nurses, midwives and other healthcare professionals. Strengthening communication and coordination among these groups will ensure a more holistic and seamless PNC experience for families. Policymakers should ensure that the health system provides adequate resources and institutional support to CCGs implementing the FCPC approach. This includes ensuring manageable caseloads, access to necessary materials and opportunities for CCGs to provide feedback on their experiences to inform policy and programme improvements.

## Conclusion

This study’s findings suggest that CCG’s knowledge of community structures and their experiences working in communities with families and community structures will bring value to implementing FCPC. Their positive attitude reflects their knowledge of the communities they serve, which is a critical component of community-based care. This study indicates that trained CCGs have the potential to implement FCPC in collaboration with other healthcare providers.
